# Comparing robust proton versus online adaptive photon radiotherapy for short-course treatment of rectal cancer

**DOI:** 10.1016/j.phro.2024.100663

**Published:** 2024-11-02

**Authors:** Johanna A. Hundvin, Unn Hege Lilleøren, Alexander Valdman, Bruno Sorcini, John Alfred Brennsæter, Camilla G. Boer, Helge E.S. Pettersen, Kathrine R. Redalen, Inger Marie Løes, Sara Pilskog

**Affiliations:** a Department of Oncology and Medical Physics, Haukeland University Hospital, Bergen, Norway; b Institute of Physics and Technology, University of Bergen, Bergen, Norway; c Department of Radiation Oncology, Karolinska University Hospital, Stockholm, Sweden; d Department of Oncology and Pathology, Karolinska Institutet, Stockholm, Sweden; e Department of Radiotherapy Physics and Engineering, Medical Physics and Nuclear Medicine, Karolinska University Hospital, Stockholm, Sweden; f Department of Physics, Norwegian University of Science and Technology, Trondheim, Norway

**Keywords:** Rectal cancer, Proton beam therapy, RAPIDO, Bowel toxicity, Online adaptation, CBCT

## Abstract

**Background and purpose:**

Image-guided proton beam therapy (IG-PBT) and cone-beam CT (CBCT)-based online adaptive photon radiotherapy (oART) have potentials to restrict radiation toxicity. They are both hypothesised to reduce therapy limiting bowel toxicity in the multimodality treatment of locally advanced rectal cancer (LARC). This study aimed to quantify the difference in relevant dose-volume metrics for these modalities.

**Material and Methods:**

Six-degrees-of-freedom IG-PBT and oART short-course radiotherapy (SCRT) were simulated for 18 LARC patients. Relative biological effectiveness (RBE) was 1.1 for IG-PBT. Delivered dose was evaluated using post-CBCTs. Target coverage was considered robust if average dose to 99% of the clinical target volume was ≥ 95% of the prescription. Organ at risk (OAR) doses were compared using dose-volume histograms and severe bowel toxicity estimated using dose–response modelling.

**Results:**

Target coverage was robust in all patients for oART and all but one patient for IG-PBT. For the main OARs, IG-PBT increased the volume exposed to ≥ 15 Gy (RBE), but reduced volumes exposed to lower doses. Both low- and high-dose exposure to bowel loops were significantly different between the modalities (median (interquartile range) IG-PBT-V_8.9Gy(RBE)_ = 92 (51–156) cm^3^, oART-V_8.9Gy(RBE)_ = 166 (107–234) cm^3^, p < 0.001; IG-PBT-V_23Gy(RBE)_ = 62 (25–106) cm^3^, oART-V_23Gy(RBE)_ = 38 (18–75) cm^3^, p < 0.001), translating into similar total grade ≥ 3 bowel toxicity risk.

**Conclusion:**

IG-PBT and oART delivered comparable and satisfying target coverage in SCRT for LARC with similar estimated risk of severe bowel toxicity. Volumes of OAR exposed to 15 Gy (RBE) or more were reduced by oART, while IG-PBT reduced the volumes receiving doses below this level.

## Highlights

• Robust proton and online adaptive photon therapy provide sufficient target coverage.
• These treatment modalities induce comparable bowel toxicity probabilities.
• Adaptive photon radiotherapy reduces doses exceeding 15 Gy (RBE) to organs at risk.

## 1 Introduction

Radiotherapy (RT) of rectal cancer is a balance between reducing the risk of local recurrences and limiting treatment complications. These complications may impact the systemic therapy. The RAPIDO trial in locally advanced rectal cancer (LARC) used short-course radiotherapy (SCRT) followed by systemic chemotherapy [1]. Compared to long course chemoradiotherapy, the trial showed a reduction in distant metastases, which has long been an important challenge in LARC [2]. This benefit came however at the cost of increased bowel toxicity, resulting in reduced doses of chemotherapy as compared to the dose prescribed by the oncologist [3].

The optimal use of radiation modality for an ideal sparing of the bowel is not yet established, and further challenged by organ motion [4], [5]. The adjacent bladder can shift the position of the target volume, and organ motion from the constant peristaltic movement and varying amount of stool and bowel gas in the digestive system can alter the basis for the dose distribution. Online adaptive photon radiotherapy (ART) is an established treatment technique, which utilises either cone-beam CTs (CBCTs) or on-couch magnetic resonance imaging (MRI) to tailor and update the radiation plan to the daily anatomy of the patient before dose delivery [6], [7]. Online ART thus eliminates the need of interfractional margins. Still, the precision of the radiation dose is limited by the transversing depth doses of photons and the remaining need of high-dose margins due to intrafractional changes, as well as target segmentation uncertainties. Proton beam therapy (PBT) with intensity modulation can create conform dose distributions with limited dose to adjacent organs at risk (OARs), allowing for non-uniform dose distributions for the individual fields. To account for the intrinsic sensitivity to density changes in the PBT beam line and setup variation, robust optimisation can be applied at the cost of a poorer conformality [8]. The range uncertainty could also be addressed by adaptive techniques, but no commercial clinical product exists for PBT online adaptation so far [9], [10], [11]. In terms of toxicity for LARC patients, several studies show reduced doses to the bowel using PBT compared to photons [12], [13]. Yet, these do not include the latter’s advantage of online adaptation, leaving the question of which treatment is most beneficial open.

As the small bowel is sensitive to radiation, sparing the bowel during RT might decrease the toxicity burden after irradiation. Therefore, our aim was to estimate the delivered dose from PBT and online ART. To further assess clinical benefits and aid treatment decision for LARC, the impact on therapy limiting acute diarrhoea was estimated.

## 2 Material and methods

### 2.1 Patient material

This study included data from 18 LARC patients (13 men, five women) with indication for preoperative RT treated by either SCRT or long-course (25 fractions) image-guided RT (IGRT) or ART at Haukeland University Hospital in 2022–2023. The Regional Committees for Medical and Health Research Ethics approved the studies before recruitment [REK402056, REK205367]. All patients had a planning-CT scan acquired in head-first supine position using Combifix^TM^ for fixation and encouraged half-full bladder technique. Intravenous contrast were applied for visualisation of tumour, lymph nodes and blood vessels. No contrast corrections were made on the CT scans for dose planning. MRI sequences (T2-weighted, diffusion-weighted) were acquired to aid target delineation.

**Fig. 1:**
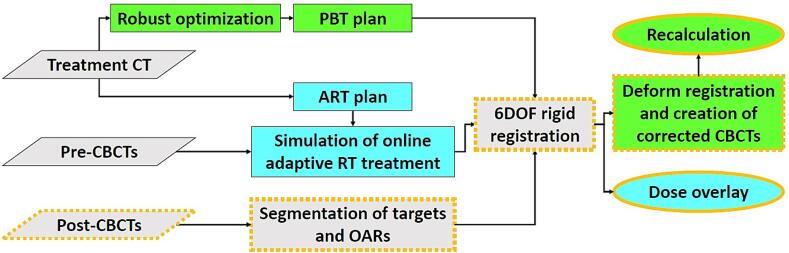
**Workflow:** Study flowchart, resulting in cone-beam CT (CBCT) dose distributions for comparative analyses. The modality specific processes are marked with green for proton beam therapy (PBT) and turquoise for online adaptive radiotherapy (ART), while the involvement of post-CBCTs is indicated with a gold line. (For interpretation of the references to colour in this figure legend, the reader is referred to the web version of this article.)

All plans created for radiation dose comparison followed the SCRT regimen, prescribing 25.0 Gy relative biological effectiveness equivalents (Gy (RBE)) in five fractions (RBE = 1.1 for PBT, RBE = 1.0 for ART) [1]. Pre-CBCT images from the first five fractions per patient were utilised for ART simulation (Fig. 1). The respective post-CBCTs were applied for dose accumulation. The post-CBCT was acquired after irradiation for the 15 patients previously treated with IGRT, and after the online adaptation planning for the three ART patients.

The clinical target volume (CTV) included the elective CTV for SCRT, delineated following the guidelines of Valentini *et al.* [14] on the planning CTs and all the daily CBCTs. The CTV included both primary and nodal gross target volumes (GTVp/GTVn), the presacral and internal iliac lymph nodes, and the complete mesorectal facia. Target delineations were made by the same researcher (J.A.H.) and subsequently verified by a senior oncologist (U.H.L.). OARs for consideration were bowel loops, bladder, pelvic bones, lumbosacral spine (LSS) and spinal canal. They were defined according to the RTOG guidelines and PRORECT (NCT04525989) protocol and guided by the open-source software TotalSegmentator [13], [15], [16], [17]. The LSS extended from the most superior vertebral body visible in the planning treatment volume or in the image volume of the post-CBCT, inferiorly to the entire sacrum, with an average volume of 295 cm^3^ on the planning CTs.

### 2.2 Planning and recalculation of PBT

**Fig. 2:**
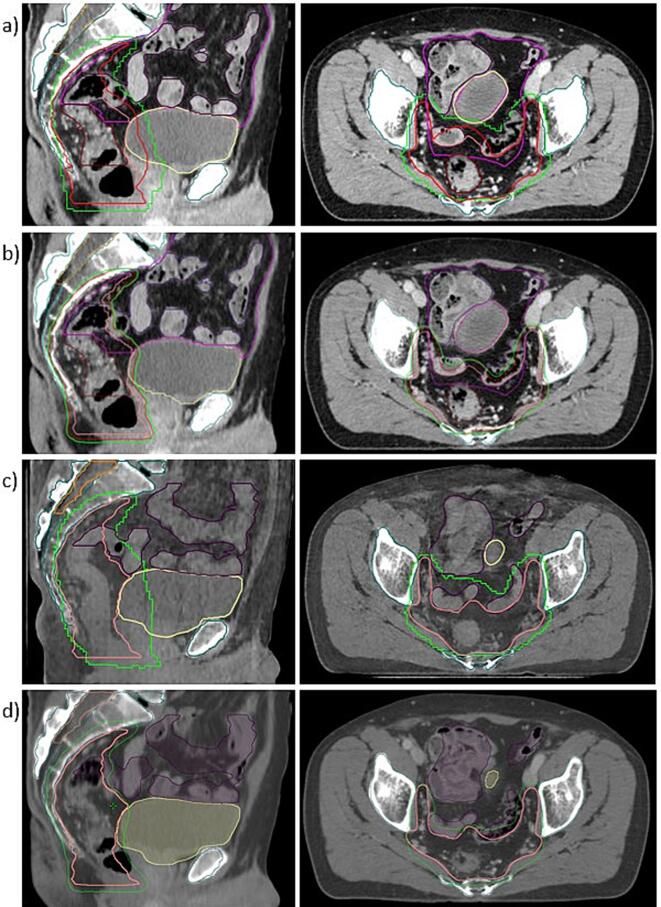
**Illustration of contours and dose distributions**: a) Proton beam therapy (PBT) plan, b) Online adaptive radiotherapy (ART) pre-treatment plan, c) Corrected cone-beam CT (CBCT) for PBT evaluation, d) Dose overlay for ART evaluation, showing structures delineated on post-CBCT and dose from the synthetic CT. Contours displayed: Gross target volume (GTV, dark red), clinical target volume (CTV, pink), robust CTV (rCTV, red; PBT only), planning target volume (PTV, red; ART only), bowel bag (magenta), bowel loops (purple), bladder (yellow), pelvic bones (dark green), spinal canal (orange), 95% isodose line (light green). Only the evaluated contours are displayed on the post-CBCTs, where lumbosacral spine (LSS) is a sub part of the pelvic bones’ structure. (For interpretation of the references to colour in this figure legend, the reader is referred to the web version of this article.)

For PBT, a robust CTV (rCTV) was created to account for varying GTV location. This was done corresponding to the PRORECT-study guidelines (Table S1). Robustly optimised intensity-modulated PBT (IMPT) plans (±3.5%, ±0.6 cm, 21 scenarios [18]) were created for a Varian ProBeam machine in RayStation 2023B (RaySearch Laboratories, Sweden), using a generic IBA beam model for planning and a Monte Carlo algorithm for dose calculation (statistical uncertainty set to 0.5%). All plans had two posterior-oblique fields (150^◦^ and 210^◦^), in accordance with the PRORECT protocol, and were optimised on CT images with bowel gas overwritten as water, avoiding hotspots beyond 110% of the prescribed dose. Plan approval required acceptable sparing of OARs and appropriate rCTV coverage as defined in Table S1 to be maintained when the dose was recalculated on the non-overwritten CT (Fig. 2a) and during the robustness test including diagonal shifts (±3.5%, ±0.6 cm, 45 scenarios).

To mimic six-degrees-of-freedom (6DoF) image guidance in PBT, the planning CTs were co-registered based on bony anatomy to the post-CBCTs. The PBT doses were subsequently recalculated on the post-CBCTs corrected to CT quality to estimate the radiation dose for each treatment fraction (Fig. 2c). The corrected CBCTs were created in RayStation 2023B, using the iterative *Corrected CBCT* algorithm, which calculates a correction map and a conversion function for the CBCT image based on the reference (planning) CT image [18], [19]. To evaluate the accuracy of the dose recalculation, all plans were also recalculated on phantom images (Figure S1).

### 2.3 Planning and simulation of online ART

For online ART, the planning target volume (PTV) was created by expanding the CTV with an isotropic margin of 4 mm. Both the pre-treatment and the daily intensity-modulated radiation therapy (IMRT) plans used 12 equidistant fields (6X-FFF), in accordance with TNT-RECORD (NCT05883800) practice, and optimising with CTV coverage of D_99.5%_>98.0% of prescribed dose (Acuros XB dose algorithm, Fig. 2b, Table S1). Criteria for plan approval were PTV dose coverage of minimum 95%, maximum 107% high dose, and limited dose to OARs with focus on bowel.

The pre-CBCTs were used as input in the online ART simulations in the Ethos research software (the Emulator, Varian, a Siemens Healthineers company), following the same workflow as the clinical system [7]. The rectum and bladder were selected as ART guiding OARs. Patient-specific goals and prioritisations were set for target coverage and low bowel dose (Table S1). The average time between pre- and post-CBCT for IGRT and ART treatments were 7 min (5–11 min) and 13 min (6–22 min), respectively. The structures from the 6DoF bone-matched post-CBCT were transferred to the synthetic CT for evaluation of the overlaid doses in the Eclipse treatment planning system (Varian, a Siemens Healthineers company) (Fig. 2d).

### 2.4 Evaluation and statistical analysis of the delivered dose

The total treatment dose was analysed as the dose metric sum of each individual treatment. The treatment was considered robust if the average D_99%_ to the CTV exceeded 95% on the post-CBCTs. The overall OAR dose was evaluated by dose-volume histograms (DVHs). Relevant dose- and volume metrics were extracted, based on the study by Holyoake *et al.* [20] where both low- and high-dose levels were related to grade ≥ 3 diarrhoea. The volume receiving 10 Gy (RBE) (V_10Gy(RBE)_) was the strongest toxicity risk predictor, and for high dose 30 Gy (RBE) (V_30Gy(RBE)_) was found significantly correlated with bowel toxicity [20]. As these dose–response models are based on conventional fractionation, the V_10Gy(RBE)_ parameter was translated to V_8.9Gy(RBE)_ and V_30Gy(RBE)_ to V_23.0Gy(RBE)_ with equivalent dose calculation (EQD2) using α/β = 10 Gy. The CTV homogeneity- and conformity index [21], maximum dose, and minimum dose from both PBT and ART planning CTs were assessed for plan-quality comparison with the PRORECT study (Table S2).

The results of the two treatment modalities were analysed in RStudio (version 2022.12.0), using median with interquartile range (IQR) and Wilcoxon signed-rank test for comparison of specific dose levels. The results were considered statistically significant if p < 0.05.

## 3 Results

**Fig. 3:**
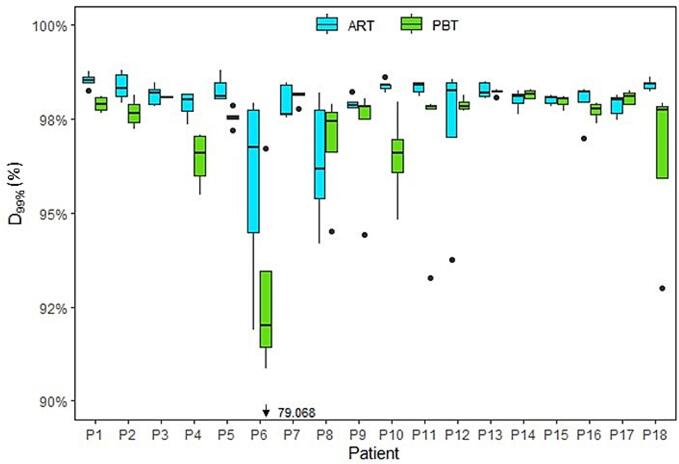
**Target coverage:** Clinical target volume (CTV) dose coverage in short-course radiotherapy (SCRT) for image-guided proton beam therapy (PBT) and online adaptive radiotherapy (ART) per patient. Median (solid line) with the 25th and 75th percentiles (box) and outliers (solid circles).

**Fig. 4:**
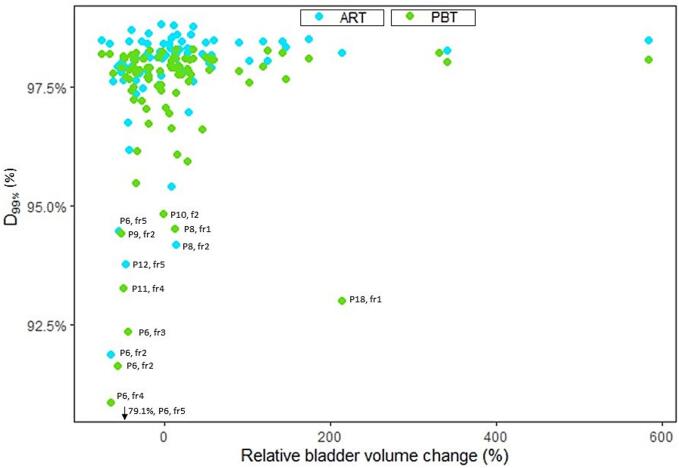
**Bladder volume influence on target coverage:** Clinical target volume (CTV) coverage (D99%) vs relative difference in bladder volume from CT to given fraction. Outliers with less than 95% target coverage are marked with patient (P) and fraction (fr).

The robustness criterium was fulfilled for all patients with online ART and all but one patient for image-guided PBT (Fig. 3). The median CTV_D99%_ was 98.0% (range 95.8%–98.5%) for online ART whereas it was 97.7% (range 90.1%–98.2%) for PBT across all patients. In 26% (9/34) of the fractions with a bladder volume reduction of more than 40% compared to the planning CTs, the CTV_D99%_ was less than 95.0% (Fig. 4), representing nine of the 13 fractions with insufficient target coverage.

**Fig. 5:**
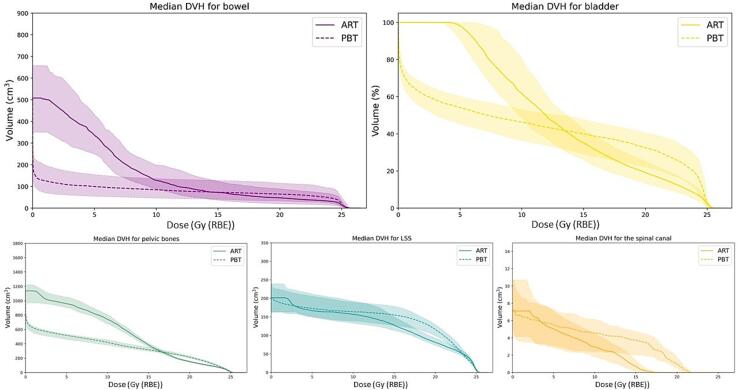
**Dose to the main organs at risk:** Median dose-volume histograms (DVHs) with quartiles (solid line: online adaptive radiotherapy (ART), dotted line: proton beam therapy (PBT)) of bowel loops, bladder, pelvic bones, lumbosacral spine (LSS), and spinal canal from treatment simulations (PBT: recalculated, ART: dose-overlay). Given in relative volume for bladder and absolute volumes for the remaining organs at risk. See Supplementary Material (Figure S2) for planning dose reference.

**Fig. 6:**
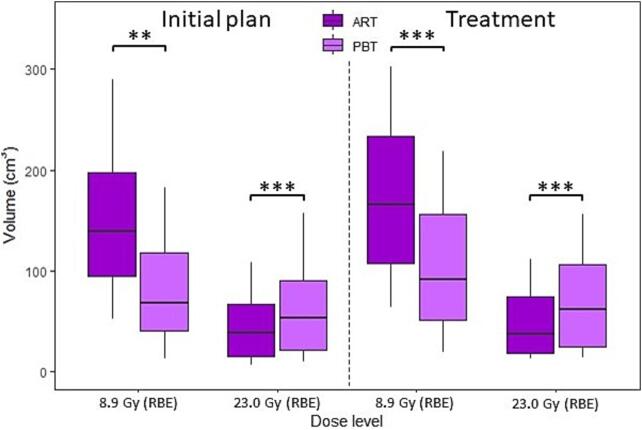
**Low and high dose boxplot:** Volume of bowel loops receiving 8.9 Gy (RBE) and 23.0 Gy (RBE) on planning CT (left) and averaged per patient over the course of treatment (right) using short-course radiotherapy (SCRT) with 25 Gy (RBE) in 5 fractions. Median (solid line) with the 25th and 75th percentiles (box) from the online adaptive radiotherapy (ART) and proton beam therapy (PBT) treatments. The significance level from Wilcoxon signed rank test is indicated with stars (**: p < 0.01, ***: p < 0.001).

The estimated delivered dose to the bowel and bladder both showed reduced exposures from PBT for doses lower than around 14 Gy (RBE), while increased for doses exceeding this level (Fig. 5). For the bowel, differences seen at planning were maintained at treatment. Statistically significant differences in treatment dose were found in favour of PBT for bowel low-dose exposure (median (IQR) PBT-V_8.9Gy(RBE)_ = 92 (51–156) cm^3^, ART-V_8.9Gy(RBE)_ = 166 (107–234) cm^3^, p < 0.001), while in favour of ART regarding high dose (median (IQR) PBT-V_23Gy(RBE)_ = 62 (25–106) cm^3^, ART-V_23Gy(RBE)_ = 38 (18–75) cm^3^, p < 0.001) (Fig. 6). Using the dose–response model, this implied 3 percentage points (p.p.) reduced probability of grade ≥ 3 bowel toxicity using PBT regarding the low dose, while an increase of about 2 p.p. with respect to ART concerning the high dose (Table S3). PBT exposed smaller volumes with doses less than 17 Gy (RBE) for the pelvic bones, and less than around 3 Gy (RBE) for LSS and the spinal canal, while ART spared tissue for doses exceeding these levels (Fig. 5).

## 4 Discussion

The OAR volume exposed to low dose was reduced by PBT, while online ART was beneficial regarding higher doses. These findings were based on dose estimation on daily imaging, although the benefits of the techniques were generally seen already from the planning CTs. This suggests feasibility of modality selection despite the known challenge of organ motion and can have implications for both treatments where SCRT is followed by chemotherapy and for dose-escalation in SCRT.

By approximating the bowel toxicity using the fractionation corrected V_8.9Gy(RBE)_ parameter in the dose–response model of long-course RT [20] and the average post-CBCT bowel volume, the risk of toxicity grade ≥ 3 could potentially be reduced from approximately 14% to 11% when choosing PBT over ART across all patients concerning low dose (Table S3). On the other hand, ART provided a risk reduction from about 18% to 16% with respect to high dose, narrowing the total risk difference to only 1 p.p. Noteworthy, this was a very rough estimation, as the dose–response model is based on treatment data of concurrent chemoradiation, which increases the risk of severe bowel toxicity compared to using RT alone [22]. Still, the toxicity risk of the total presurgical therapy should be considered for the RAPIDO regimen, as the challenging acute bowel toxicity was seen during chemotherapy, not during the preceding SCRT. Uncertainties are also introduced by differences in segmentation, as the model applied considers only the small bowel while this study combined the evaluation of both small bowel and colon loops. Though, as shown by Banerjee *et al*. [23], both delineated small bowel loops and the peritoneal space (including the large bowel) can be utilised to predict grade ≥ 3 toxicity.

Introducing online adaptation to the IMRT plans, the previously known benefit in dose- and volume metrics from PBT was reduced. This was seen in the median or average crossover between the treatment modalities from approximately 23 Gy (RBE) for bladder and 20 Gy (RBE) for bowel in the work on SCRT (5 Gy (RBE)/5 fr) of Pedone et al. [13], to around 14 Gy (RBE) using ART. In the SCRT study by Jeans *et al.,* the total bowel volume receiving 15 Gy (RBE) was around 60 cm^3^ using protons, which was lower than for both treatment modalities in our study (Fig. 5) [24]. This could be partly attributable to slight differences in bowel segmentations and also reflect a potential benefit of their use of other uncertainty parameters of PBT (5 mm, 5%). Colaco *et al.* simulated 3D-conformal RT, IMRT and single-field PBT for long course-RT of rectal cancer [25]. The results were comparable to our study, as the high-dose volume was smaller with IMRT, even without any adaptation.

Validated SCRT toxicity data for the bladder is scarce, though probably not a clinical issue considering the dose constraints of conventional fractionation at much higher levels [26]. Regarding hematologic toxicity, Huang et al. recommended maintaining LSS V_10Gy(RBE)_ < 87% to avoid grade two or higher, equivalent to on average 257 cm^3^ in our study, though based on concurrent chemoradiotherapy data with RT doses of 1.8 Gy per fraction [27]. The delineation of the spinal canal was dependent on the length on the CBCT scan and often very small, leading to ambiguous analysis. Despite its proximity to the target, and location in the entrance dose of the PBT, the toxicity does not seem to be a clinical issue, with no case of acute lumbosacral plexopathy in the first 40 PRORECT patients [28]. To strengthen our study, we could have included the linear energy transfer (LET) in our considerations. High LET ideally distributed in the target volume might improve local control, while high LET mistakenly disposed in the bowel loops trailing the CTV by posterior fields might increase the toxicity burden.

An occasional greater variation of the treatment doses in PBT was expected due to the interfractional motion being potentially larger than intrafractional motion over a short timespan [29], [30]. Our data indicated that care should be taken for larger reductions in bladder volume compared to the planning CT, as the possible increase in target mobility for certain patients jeopardises the target coverage. This was especially seen in the under-dosage fractions of patient 6, a woman with an interfractional reduction in bladder volume ranging from 44%–65%. Notably, the patient’s extreme outlier was due to a clear shift in target position, which in a clinical setting with PBT would be apparent on the pre-CBCT and actions taken thereafter before irradiation.

The strength of this study was the use of post-CBCT to approach the actual delivered dose instead of the planned dose. However, the results were highly dependent on the quality of the scans, where especially the bowel was prone to artefacts originating from bowel gas, leaving the delineated volume more extensive and uncertain than intended. Further, the corrected CBCT is not yet validated for clinical proton dose calculation. Still, our phantom verifications showed minor dose deviations compared to the results (Figure S1). The study’s concordance with ongoing clinical trials was beneficial in terms of impending clinical outcomes to validate the results. Yet, the practices were not identical, and the importance of standardised bladder-filling protocols has become particularly apparent. As our patients on average had a smaller and less consistent bladder volume than the first patients included in the PRORECT study [13], the risk of impaired target coverage may have been overestimated. In addition, we utilised multifield optimisation (MFO) IMPT, in contrast to single-field uniform dose optimisation (SFUD). Even if using SFUD does not ensure a robust plan, the possibly sharp in-field gradients of a MFO plan may seriously degrade plan quality in the face of the anatomical changes common in the pelvic region (*e.g.,* gas) [31]. Still, we reckon that the robust optimisation outweighs the loss of robustness without SFUD and potentially gives more freedom in dose adjustment to target volume. The optimisation flexibility could have been increased by using an arc or adding more treatment fields, the latter tested during the PRORECT study [13]. Arc is still not widely available, and we chose to stay with the PRORECT practice [13], [32].

The TNT-RECORD-study protocol has no rigidly defined PTV margin for ART. The 4 mm margin was verified as sufficient in this and previous work [33], where a further reduction was considered unsafe due to target delineation uncertainty. The isotropic 6 mm margin in the robust optimisation of the PBT plans was taken directly from the PRORECT protocol [13]. In principle, the PBT treatment could be considered an adaptive treatment where the large margins eradicated the need of adaption in 17 of the 18 patients. Following the Danish guidelines of 5 mm and 3.5% for reirradiation of rectal cancer (NCT04695782) or optimising this margin further could decrease the difference in excessive high dose to bowel and bladder but may come at a cost of an increased call for adaption. Further, the average time interval of only seven minutes between the utilised CBCT was rather short compared to the potential span of an adaptive treatment, which is normally around 15 min in our clinic, and may have led to an underestimation of the intrafractional motion.

In conclusion, both 6DoF image-guided PBT and CBCT-based online ART delivered comparable and satisfying target coverage in the SCRT regimen for LARC. PBT required pre-CBCT supervision of potential dose distribution mainly due to bladder filling. For the main OARs, PBT spared larger volumes exposed to approximately 15 Gy (RBE) or less, whereas online ART had the potential to spare volumes receiving higher doses.

## CRediT authorship contribution statement

**Johanna A. Hundvin:** Investigation, Methodology, Formal analysis, Data curation, Visualization, Writing – original draft, Writing – review & editing. **Unn Hege Lilleøren:** Validation, Resources, Data curation, Writing – review & editing. **Alexander Valdman:** Validation, Writing – review & editing. **Bruno Sorcini:** Validation, Writing – review & editing. **John Alfred Brennsæter:** Resources, Writing – review & editing. **Camilla G. Boer:** Validation, Writing – review & editing. **Helge E.S. Pettersen:** Software, Writing – review & editing. **Kathrine R. Redalen:** Conceptualization, Writing – review & editing, Supervision. **Inger Marie Løes:** Validation, Writing – review & editing, Supervision. **Sara Pilskog:** Conceptualization, Data curation, Methodology, Project administration, Writing – review & editing, Supervision, Funding acquisition.

## Declaration of competing interest

The authors declare the following financial interests/personal relationships which may be considered as potential competing interests: J.A.H. is funded by a grant from Varian, a Siemens Healthineers company (grant holder S.P.). S.P. is funded by Norwegian cancer society, grant number 223048–2021. Neither Varian nor the Norwegian cancer society had any influence on study design, results, or conclusion of the study.
