# Supplementary material for: Comparing robust proton versus online adaptive photon radiotherapy for short-course treatment of rectal cancer
Source: Phys Imaging Radiat Oncol. 2024 Nov 2;32:100663. doi: 10.1016/j.phro.2024.100663 (PMC11570970; doi:10.1016/j.phro.2024.100663)
Supplement: Supplementary Data 1 [file mmc1.pdf]

Supplementary material

Table S1*: Initial planning directives for proton beam therapy (PBT) and online adaptive photon radiotherapy (ART), organ at risk (OAR) constraints and target coverage criteria. The Bowel bag structure was only included on the planning CTs for optimization. The bowel was delineated as the bowel loops on all images, except for areas where air artefacts forced the contouring closer to a bowel-bag structure. The patient-averaged bowel volume on post-cone-beam-CT was 574 cm^3 (range 203–1574 cm^3). GTV: Gross target volume, PTV: planning target volume, rCTV: robust clinical target volume, corresponding to the PRORECT-study guidelines, i.e. the smoothed combination of the CTV and GTVs with margins ($GTVp_{Radial\ margin}$ = 15 mm, $GTVp_{Craniocaudal\ margin}$ = 20 mm, $GTVn_{Isotropic\ margin}$ = 10 mm) cropped towards muscles and bones, but not towards the bladder.*

		Structure	Type	Weight	Description			
Initial planning directives	PBT	GTV	Objective	1900	Uniform dose 25.50 Gy (RBE), Robust			
		rCTV	Objective	500	Uniform dose 25.00 Gy (RBE), Robust			
		BODY	Objective	1500	Max dose 26.00 Gy (RBE), Robust			
		BODY	Objective	10	Dose fall-off [H] 25.00 Gy (RBE) [L] 5.00 Gy (RBE), Low dose distance 1.20 cm			
		Structure	Priority		Goal			Accepted variation
	ART	PTV	P1		$D_{98\%}$	≥	95%	≥ 94%
			P2		$D_{0.1\ cm3}$	<	105%	< 107%
		CTV	P2		$D_{99.5\%}$	>	98%	≥ 95%
		Bowel bag	P2		$V_{7.5\ Gy\ (RBE)}$	<	450 cm^3	< 500 cm^3
			P2		$V_{20\ Gy\ (RBE)}$	<	100 cm^3	< 150 cm^3
		Bladder	P3		D_{mean}	≤	20 Gy (RBE)	≤ 22 Gy (RBE)
OAR planning constraints		Structure	Priority		Dose constraint			
		Bowel bag	2		$V_{18\ Gy\ (RBE)}$	≤	450 cm^3	
		Femoral heads	2		D_{mean}	<	25 Gy (RBE)	
		Spinal canal	2		$V_{25\ Gy\ (RBE)}$	<	60%	
		Pelvic bones	3		ALARA, avoid hotspots			
		Bladder	3		ALARA, avoid hotspots			
Target volume criteria		Evaluation	Target		Coverage criteria			
	PBT	Robustness criteria	rCTV: All test cases		$D_{90\%}$	≥	95%	
			rCTV: For at least 43 of 45 robustness test cases		$D_{98\%}$	≥	95%	
			GTV: All test cases		$D_{95\%}$	≥	100%	
			GTV: For at least 43 of 45 robustness test cases		$D_{98\%}$	≥	100%	
	ART	Pre-treatment plan	PTV		$D_{0.1cm^3}$	≤	107%	
			PTV		$D_{98\%}$	≥	95%	

Table S2: *Patient and plan characteristics to facilitate comparison of treatment plan quality with the ongoing PRORECT study; volume (V), homogeneity index (HI), conformity index (CI), minimum dose (D_{min}), and maximum dose (D_{max}). Target volumes: Gross tumour volume (GTV), clinical target volume (CTV), robust CTV (rCVT), planning target volume (PTV).*

Patient	Gender	Planning CT				PBT (rCTV)				ART (PTV)			
		V_{GTV} (cm^3)	V_{CTV} (cm^3)	V_{rCTV} (cm^3)	V_{PTV} (cm^3)	HI_{rCTV}	CI_{rCTV}	D_{min} (%) ($D_{99.5\%}$)	D_{max} (%) ($D_{0.1\ cm3}$)	HI_{PTV}	CI_{PTV}	D_{min} (%) ($D_{99.5\%}$)	D_{max} (%) ($D_{0.1\ cm3}$)
1	Female	25	586	679	906	0.03	1.0	98	103	0.06	1.0	95	105
2	Male	78	735	809	1061	0.04	1.0	98	103	0.05	1.0	96	104
3	Male	61	644	685	939	0.04	1.0	98	103	0.05	1.0	96	105
4	Female	46	659	733	1002	0.04	1.0	95	105	0.06	1.0	96	105
5	Male	160	792	831	1104	0.03	1.0	98	103	0.05	1.0	96	104
6	Female	60	616	729	920	0.04	1.0	98	103	0.06	1.0	96	104
7	Male	30	665	732	941	0.03	1.0	98	103	0.05	1.0	96	105
8	Male	60	588	638	851	0.04	1.0	96	103	0.06	1.0	96	105
9	Male	151	1069	1217	1374	0.04	1.0	98	103	0.05	1.0	96	105
10	Male	99	670	752	925	0.04	1.0	98	103	0.05	1.0	96	104
11	Male	105	526	631	797	0.04	1.0	98	103	0.05	1.0	96	105
12	Male	56	668	753	955	0.04	1.0	98	103	0.06	1.0	96	104
13	Male	68	669	745	962	0.03	1.0	98	103	0.05	1.0	96	105
14	Female	90	829	876	1172	0.03	1.0	98	103	0.05	1.0	96	105
15	Female	97	609	746	938	0.04	1.0	97	103	0.06	1.0	96	105
16	Male	68	508	594	740	0.04	1.0	98	103	0.06	1.0	98	105
17	Male	17	438	472	683	0.03	1.0	98	103	0.06	1.0	98	104
18	Male	89	594	654	845	0.04	1.0	98	103	0.05	1.0	96	104

Table S3: *An estimation of clinical impact of the dose differences through the dose-response model of Holyoake et al. with initial and average treatment values from all patients in total and examples of individual patient benefit (patient 3 and patient 9) using proton beam therapy (PBT) or online adaptive radiotherapy (ART). Difference in normal tissue complication probability (NTCP) is given as ΔNTCP = NTCP(PBT) -NTCP(ART).*

NTCP model

$$NTCP = \frac{1}{1 + e^{-s}} \qquad s = b_0 + b_1 \cdot V_{x\,Gy}$$

			Examples								
			ALL			*P3*			*P9*		
$V_{X\ Gy\ (RBE)}$	b_0	b_1	*NTCP PBT*	*NTCP ART*	*ΔNTCP*	*NTCP PBT*	*NTCP ART*	*ΔNTCP*	*NTCP PBT*	*NTCP ART*	*ΔNTCP*
			Initial plan			*Initial plan*			*Initial plan*		
10	*-2.63*	*0.005*	*10%*	*13%*	*-3%*	*10%*	*16%*	*-6%*	*11%*	*19%*	*-8%*
30	*-2.01*	*0.007*	*17%*	*15%*	*2%*	*16%*	*15%*	*1%*	*18%*	*15%*	*3%*
			Treatment			*Treatment*			*Treatment*		
10	*-2.63*	*0.005*	*11%*	*14%*	*-3%*	*8%*	*15%*	*-7%*	*17%*	*23%*	*-6%*
30	*-2.01*	*0.007*	*18%*	*16%*	*2%*	*14%*	*14%*	*0%*	*23%*	*15%*	*8%*

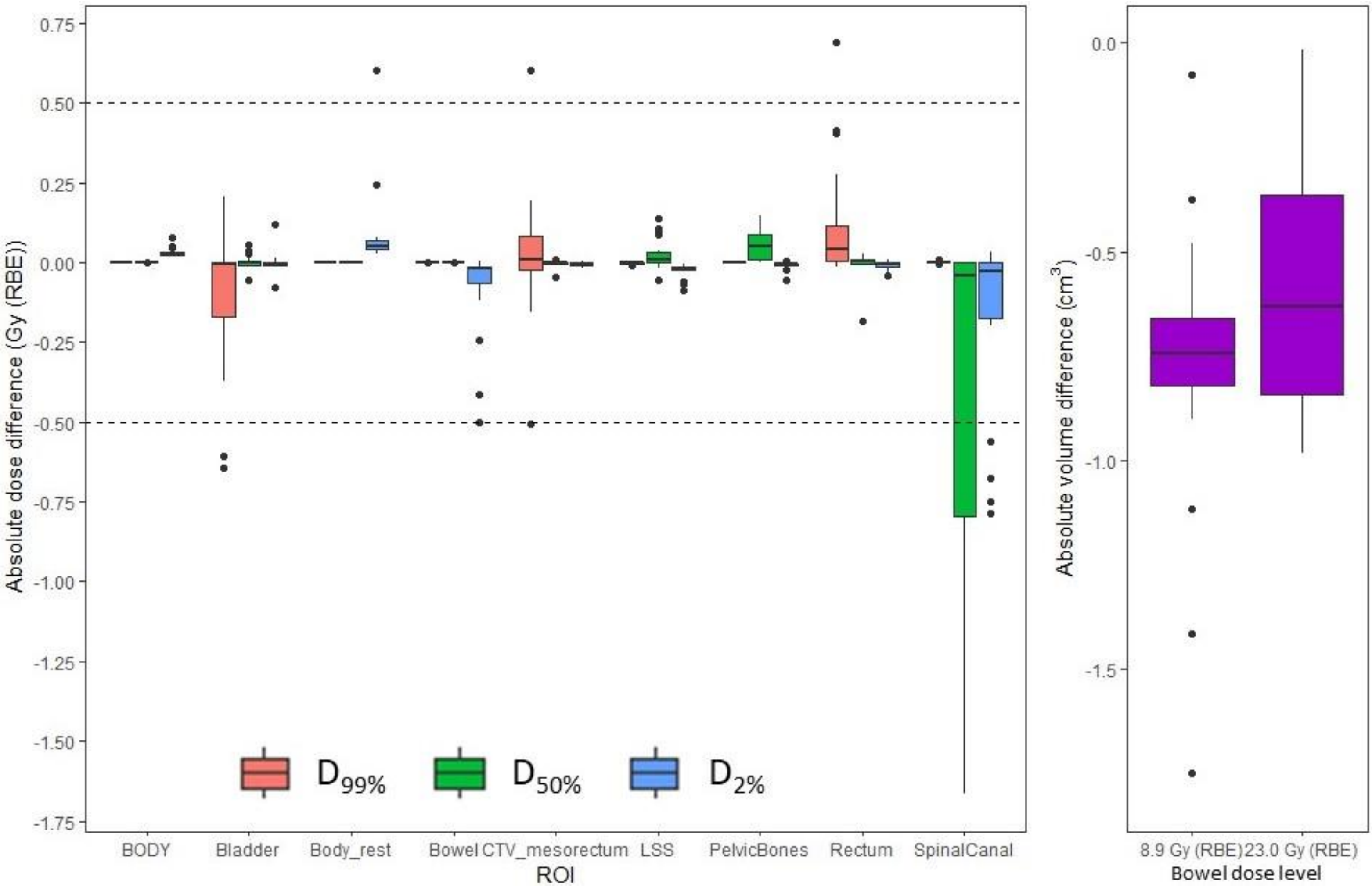

Figure S1: *Control of corrected cone-beam CT (CBCT) doses. As the corrected CBCT is not yet validated for clinical use for protons, all proton treatment plans were recalculated on a centre-aligned CT image of a pelvic phantom (CIRS, Sun Nuclear) and its six-degrees-of-freedom-matched corrected CBCT (generic IBA beam model, Monte Carlo algorithm for dose calculation with 0.5% uncertainty). The differences in dose distribution are given in absolute dose (left) and volume (right). The regions of interest (ROI) are the bladder, BODY, bowel, lumbosacral spine (LSS), pelvic bones, rectum, spinal canal, and a generic mesorectum CTV structure. An additional structure encompassing the BODY when all the other ROIs were subtracted were included to capture more of the deviation in-between delineated structures. The size of the phantom bladder (55 cm^3) and spinal canal (12 cm^3) in addition to their proximity to gradients makes them especially sensitive to small deviations in these metrics.*

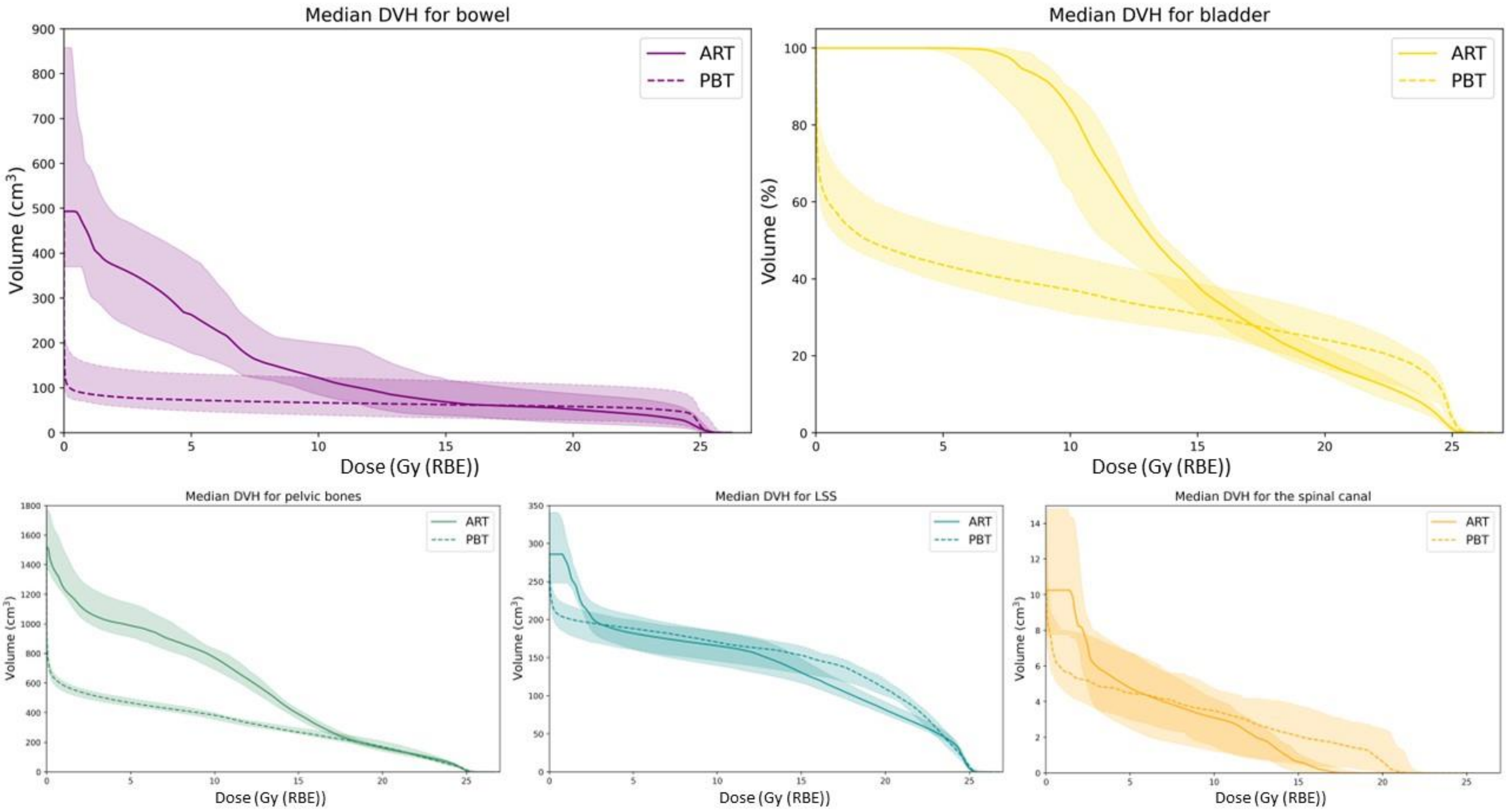

Figure S2: *Dose to the main organs at risk (OARs) on planning CT. Median dose-volume histograms (DVHs) with quartiles (solid line: online adaptive radiotherapy (ART), dotted line: proton beam therapy (PBT)) of bowel loops, bladder, pelvic bones, lumbosacral spine (LSS), and spinal canal on the planning CT for reference of initial plan quality. Given in relative volume for bladder and absolute volumes for the remaining OARs.*
